# Supplementary material for: Molecular insight into Aspergillus oryzae β-mannanase interacting with mannotriose revealed by molecular dynamic simulation study
Source: PLoS One. 2022 Sep 16;17(9):e0268333. doi: 10.1371/journal.pone.0268333 (PMC9480991; doi:10.1371/journal.pone.0268333)
Supplement: S1 Table — (DOCX) [file pone.0268333.s001.docx]

**Supporting Table S1.** Interaction of *βManAo* amino acids with substrate (M3) involved in different chemicals bonds

| **Docking Complex** | **Type of Chemical bond** | **Amino acids involved** |
| --- | --- | --- |
| *βManAo-*M3 | Conventional H-bond | Glu208 (2 bonds), Asn151, Glu244 (2 bonds) |
|  | Van der Waals interaction | Asn207, Trp95, Tyr156, Asp155, Arg210, Phe246, Tyr256, Trp287, Tyr283, Ser286, Trp153 |
