# Supplementary material for: Molecular insight into Aspergillus oryzae β-mannanase interacting with mannotriose revealed by molecular dynamic simulation study
Source: PLoS One. 2022 Sep 16;17(9):e0268333. doi: 10.1371/journal.pone.0268333 (PMC9480991; doi:10.1371/journal.pone.0268333)
Supplement: Attachment — Submitted filename: Reviewers Reply.doc [file pone.0268333.s002.doc]

**Journal Requirements:**

1. Please ensure that your manuscript meets PLOS ONE's style requirements, including those for file naming. The PLOS ONE style templates can be found at 
https://journals.plos.org/plosone/s/file?id=wjVg/PLOSOne_formatting_sample_main_body.pdf  and https://journals.plos.org/plosone/s/file?id=ba62/PLOSOne_formatting_sample_title_authors_affiliations.pdf

**Reply: Revised as suggested**

2.  Please include your tables as part of your main manuscript and remove the individual files. Please note that supplementary tables (should remain/ be uploaded) as separate "supporting information" files.

**Reply: Revised as suggested**

3. We note that you have stated that you will provide repository information for your data at acceptance. Should your manuscript be accepted for publication, we will hold it until you provide the relevant accession numbers or DOIs necessary to access your data. If you wish to make changes to your Data Availability statement, please describe these changes in your cover letter and we will update your Data Availability statement to reflect the information you provide.

**Reply:** No, All relevant data are within the paper and its Supporting Information files.

4. Thank you for stating the following in the Acknowledgments Section of your manuscript: 
(Author UKJ is grateful to ICMR, New Delhi for providing financial assistance as Senior Research Fellow. Authors thank Sophisticated Instrumentation Centre (SIC), Dr. Harisingh Gour Vishwavidyalaya, Sagar and DST PURSE (II) scheme for instrumentation facilities and financial support. Authors acknowledge the HPC facility of Indian Institute of Technology, Delhi for providing the computational resources for running the MD simulations.) We note that you have provided funding information that is not currently declared in your Funding Statement. However, funding information should not appear in the Acknowledgments section or other areas of your manuscript. We will only publish funding information present in the Funding Statement section of the online submission form. Please remove any funding-related text from the manuscript and let us know how you would like to update your Funding Statement. Currently, your Funding Statement reads as follows: (The author(s) received no specific funding for this work.)

Please include your amended statements within your cover letter; we will change the online submission form on your behalf.

**Reply:**

The funding information regarding ‘*financial* assistance as Senior Research Fellow to UKJ*’* has now been removed from the Acknowledgments section.

5. Please review your reference list to ensure that it is complete and correct. If you have cited papers that have been retracted, please include the rationale for doing so in the manuscript text, or remove these references and replace them with relevant current references. Any changes to the reference list should be mentioned in the rebuttal letter that accompanies your revised manuscript. If you need to cite a retracted article, indicate the article’s retracted status in the References list and also include a citation and full reference for the retraction notice.

 **Reply:** Not applicable

**Reviewers' comments:**

Reviewer's Responses to Questions

**Comments to the Author**

1. Is the manuscript technically sound, and do the data support the conclusions?

The manuscript must describe a technically sound piece of scientific research with data that supports the conclusions. Experiments must have been conducted rigorously, with appropriate controls, replication, and sample sizes. The conclusions must be drawn appropriately based on the data presented.

Reviewer #1: Yes

Reviewer #2: Yes

**Reply:** We are thankful to the reviewers for their kind comments.

2. Has the statistical analysis been performed appropriately and rigorously?

Reviewer #1: No

Reviewer #2: Yes

**Reply:** We have again checked the requirement for statistical analysis, where relevant.

3. Have the authors made all data underlying the findings in their manuscript fully available?

Reviewer #1: Yes

Reviewer #2: Yes

**Reply:** We are thankful to the reviewers for their kind comments.

4. Is the manuscript presented in an intelligible fashion and written in standard English?

PLOS ONE does not copyedit accepted manuscripts, so the language in submitted articles must be clear, correct, and unambiguous. Any typographical or grammatical errors should be corrected at revision, so please note any specific errors here.

Reviewer #1: Yes

Reviewer #2: No

**Reply:** We are thankful to the reviewers for their kind comments. As per the comment of Reviewer #2, we have now revised the manuscript thoroughly with the help of a native English speaker.

5. Review Comments to the Author

Please use the space provided to explain your answers to the questions above. You may also include additional comments for the author, including concerns about dual publication, research ethics, or publication ethics. (Please upload your review as an attachment if it exceeds 20,000 characters)

**Reviewer #1:**

The manuscript by Naveen Kango et al. entitled “Molecular insight into *Aspergillus* *oryzae* β-mannanase interacting with mannotriose revealed by molecular dynamic simulation study” reports an in-silico approach to study structural and functional aspects of an *Aspergillus oryzae* β- mannanase (*βManAo*). Fungal β-mannanases, hydrolyze β-1, 4-glycosidic bonds of mannans and find application in the generation of mannose and prebiotic mannooligosaccharides (MOS). The authors, through homology modeling and secondary structure prevision, created a *βManAo* model, that was validated and used for the docking, molecular dynamics, and secondary structure prevision studies. From the results obtained, were identified some features of this enzyme, like the binding site, the most important amino acid (Glu244 was found to play a key role in interaction with mannotriose, indicating conserved amino acid for the catalytic reaction), A detailed PCA analysis revealed the very flexible nature of the protein and energy landscapes suggested high conformation sub-states and complex dynamic behavior of the protein. DSSP analysis revealed the major transition of the protein from helix to turn when binding the mannotriose. The authors conclude that a hypothetical model was created, the binding mechanism of *A. oryzae* β-mannanase with mannotriose was identified where the Glu208 and Glu244 are the key amino acids at the active binding site, and also other amino acids (Asn207, Asp155, and Ser286) are important for the binding with the mannotriose.

**Reply:** We are thankful to the reviewers for their kind comments.

The manuscript in the present form demands a revision before it can be published, so this reviewer suggests a “minor revision” of the paper.

Major issue:

1) The abstract is not very attractive; it should arouse curiosity in the reader and should be clearer. It should include details about the methodologies used to reach the results and in brief some details about the results in terms of numbers.

**Reply:** We thank the reviewer for this very important comment. We have now revised the abstract in order to make it clearer and more interesting for the reader. We have also included details about the methodologies used and provided some brief details of results in numbers.

2) The results sub-section about the docking should be extended.

**Reply:** As suggested, the results sub-section about the docking has now been extended. The extended section is now named as Section 2.5 and Section 2.6.

3) About the conclusion section: it is too simple, in my opinion, needs some improvements, as well as, add all the results obtained and a small perspective paragraph.

**Reply:** We have revised the conclusion and added some results, as well as a perspective paragraph. Section 4.

Minor issue:

1) Please try to improve, in general, the readability of the text helps the reader to better understand the paper.

**Reply:** We have revised the text thoroughly so as to improve the readability of the paper.

2) All paper needs an accurate English revision and an accurate formatting check.

**Reply:** We have revised the text in terms of the use of the English language and formatting as suggested.

3) In Figure 1, panels A and E should be improved in terms of quality.

**Reply:** As suggested, the Figure 1, panels A and E are improved in terms of quality now.

**Reviewer #2:**

In this study, the authors show their results, using in silico approach of structural and functional aspects of an *Aspergillus* *oryzae* β- mannanase (*βManAo*). The manuscript is the interest to the journal, but I suggest publishing after minor revision” of the paper. In particular:

**Reply:** We thank the esteemed reviewer for going through the manuscript and kind comments.

1. The abstract is not clear enough. I suggest the authors rewrite and improve.

**Reply:** We thank the reviewer for this comment. We have re-written the abstract to make it clearer.

2. In my opinion, are needed to improve the conclusion section of the manuscript. In particular, the authors should stress how their results can improve the know-how in the mannanase enzyme and their application in the degradation of mannanase.

**Reply:** We have re-written the conclusion section to provide know-how on the mannanase enzymes and their application in the degradation of mannan.

3. The quality of Figure 1, panels A and E, is too low. Please, the authors should provide it.

**Reply:** As suggested, the Figure 1, panels A and E are improved in terms of quality now.

4. The figure legends need more details. Please, the authors should provide it.

**Reply:** Thank you for this very important comment. The figure legends have now been revised to render them complete and informative.

5. Accurate English revision of the text is needed.

**Reply:** We have revised the English language in the text as suggested.
